# Supplementary figures and images for: Mitochondrial cytochrome c oxidase subunit I (COI) metabarcoding of Foraminifera communities using taxon-specific primers
Source: PeerJ. 2022 Sep 5;10:e13952. doi: 10.7717/peerj.13952 (PMC9454970; doi:10.7717/peerj.13952)

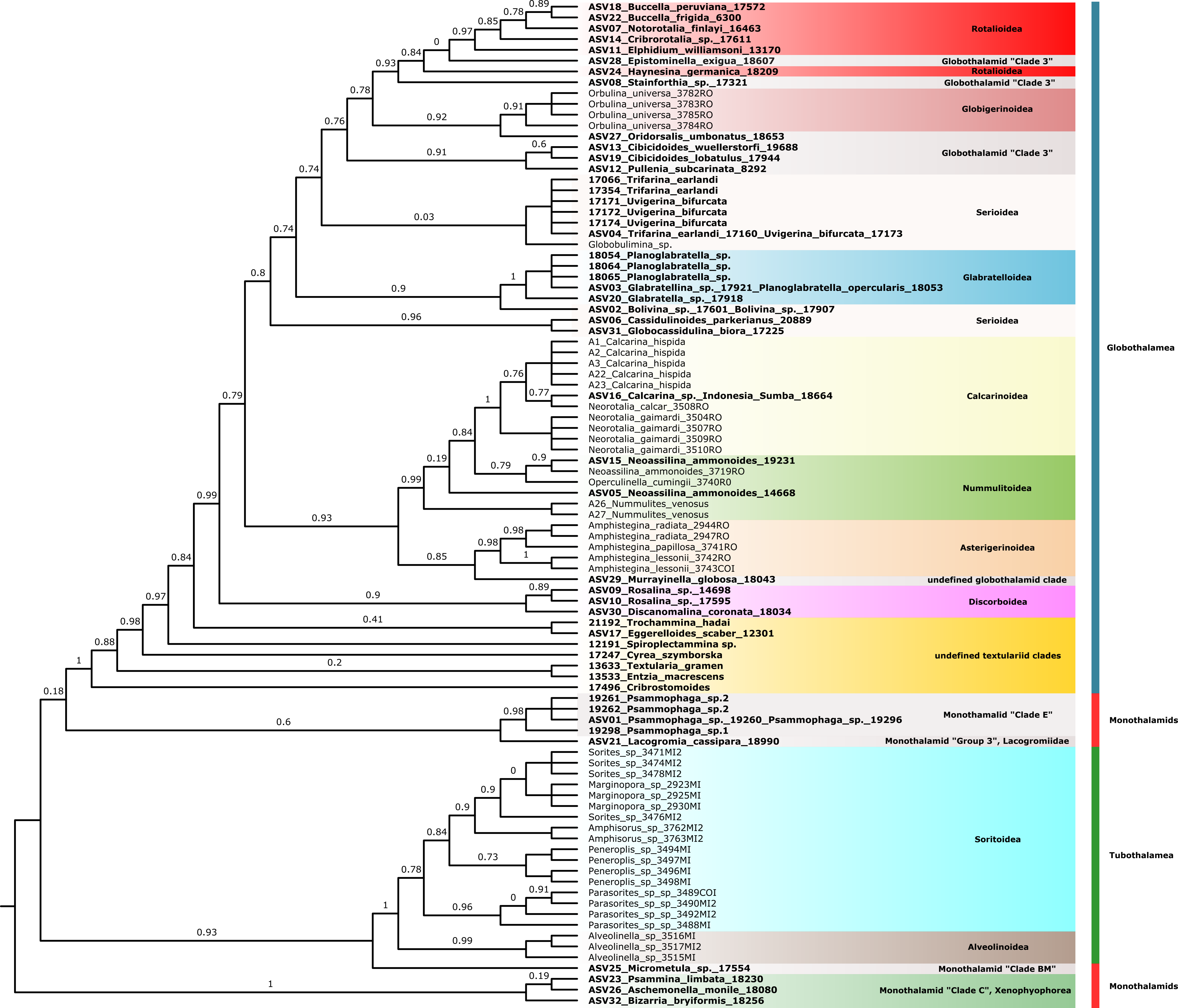

Globothalamia

Monothalamids

Tubothalamia

Monothalamids

0.08

Supplement: Supplemental Information 4 [file peerj-10-13952-s004.pdf]

# Interpolation and extrapolation of ASV richness

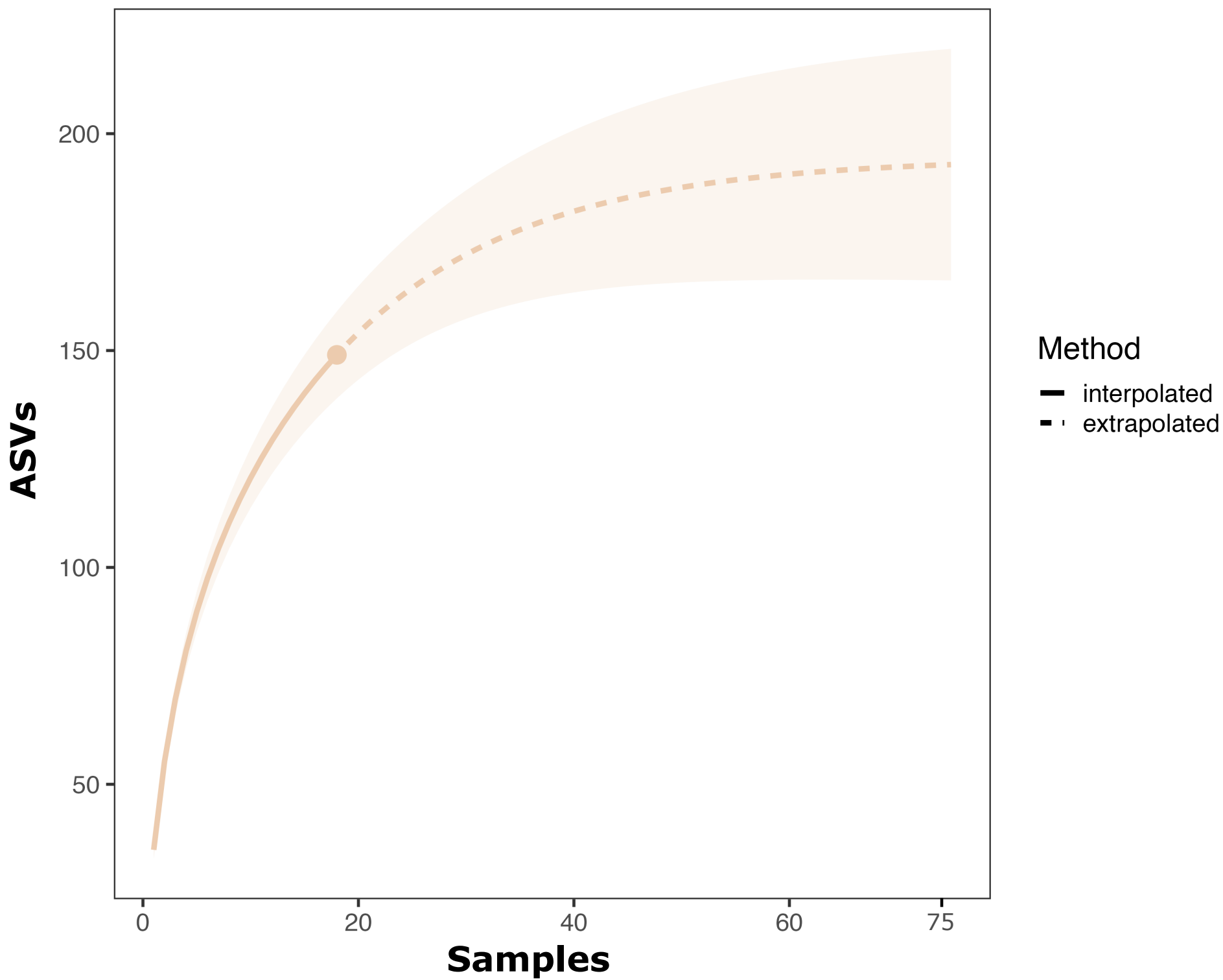

Supplement: Supplemental Information 5 — Fig. S2 Accumulation curves showing ASV richness in sediment samples amplified with Foraminifera COI primers. Solid lines show interpolated data, dashed lines show extrapolated data. [file peerj-10-13952-s005.pdf]
